# Autologous Fibrin Glue as an Encapsulating Scaffold for Delivery of Retinal Progenitor Cells

**DOI:** 10.3389/fbioe.2014.00085

**Published:** 2015-02-03

**Authors:** Tamer A. E. Ahmed, Randy Ringuette, Valerie A. Wallace, May Griffith

**Affiliations:** ^1^Vision Program, Ottawa Hospital Research Institute, Ottawa, ON, Canada; ^2^Medical Biotechnology Department, Genetic Engineering and Biotechnology Research Institute, City of Scientific Research and Technology Applications (SRTA-City), Alexandria, Egypt; ^3^Department of Cellular and Molecular Medicine, University of Ottawa, Ottawa, ON, Canada; ^4^Department of Biochemistry, Microbiology and Immunology, University of Ottawa, Ottawa, ON, Canada; ^5^Vision Science Research Program, Toronto Western Research Institute, Toronto, ON, Canada; ^6^Department of Clinical and Experimental Medicine, Integrative Regenerative Medicine Centre, Linköping University, Linköping, Sweden

**Keywords:** fibrin glue, retinal progenitor cells, encapsulation, cell delivery

## Abstract

The retina is a highly sophisticated piece of the neural machinery that begins the translation of incoming light signals into meaningful visual information. Several degenerative diseases of the retina are characterized by photoreceptor loss and eventually lead to irreversible blindness. Regenerative medicine, using tissue engineering-based constructs to deliver progenitor cells or photoreceptors along with supporting carrier matrix is a promising approach for restoration of structure and function. Fresh fibrin glue (FG) produced by the CryoSeal^®^FS system in combination with mouse retinal progenitor cells (RPCs) were evaluated in this study. *In vitro* expanded RPCs isolated from postnatal mouse retina were encapsulated into FG and cultured in the presence of the protease inhibitor, tranexamic acid. Encapsulation of RPCs into FG did not show adverse effects on cell proliferation or cell survival. RPCs exhibited fibroblast-like morphology concomitantly with attachment to the encapsulating FG surface. They expressed α7 and β3 integrin subunits that could mediate attachment to fibrin matrix via an RGD-independent mechanism. The three-dimensional environment and the attachment surface provided by FG was associated with a rapid down-regulation of the progenitor marker SOX2 and enhanced the expression of the differentiation markers cone-rod homeobox and recoverin. However, the *in vitro* culture conditions did not promote full differentiation into mature photoreceptors. Nevertheless, we have shown that autologous fibrin, when fabricated into a scaffold for RPCs for delivery to the retina, provides the cells with external cues that could potentially improve the differentiation events. Hence, transient encapsulation of RPCs into FG could be a valid and potential treatment strategy to promote retinal regeneration following degenerative diseases. However, further optimization is necessary to maximize the outcomes in terms of mature photoreceptors.

## Introduction

The retina is the highly specialized, multi-layered light-sensitive neural tissue located at the back of the eye that receives visual stimuli and converts it into chemical and electrical signals that are interpreted by the brain for sight (Inoue et al., 2010; Karl and Reh, 2010). The retina is susceptible to a range of degenerative diseases, the most common being age-related macular degeneration (AMD), diabetic retinopathy (DR), and genetic diseases such as retinitis pigmentosa (RP) (Ballios et al., 2010). Vision loss associated with AMD and RP is caused by loss of function or death of photoreceptors. Hence, most therapies for these conditions aim to prevent photoreceptor loss or restore photoreceptor function (Ballios et al., 2010). Two main routes are being explored: the replacement of the photoreceptor with electronic visual implants that transmit electrical signals to the brain (Zrenner, 2002); and cell-based therapies including retinal transplantation (Pritchard et al., 2010; Taylor et al., 2013) or transplantation of photoreceptor precursors (MacLaren et al., 2006).

Delivery of healthy autologous or allogeneic cells that have the capacity to integrate with the host retina and replace photoreceptor function to the subretinal space is a promising strategy to restore vision (Banin et al., 2006; West et al., 2008; Ballios et al., 2010; Mansergh et al., 2010). A wide range of cells types including progenitor cells, mature photoreceptors, retinal sheets, and retinal pigmented epithelial cells have been delivered to the subretinal space and show variable degrees of vision improvement in animal models (Hynes and Lavik, 2010). It has been demonstrated that delivery of retinal progenitor cell (RPC) suspensions into the subretinal space in the absence of supporting scaffold results in insufficient cell survival (Tomita et al., 2005; Ballios et al., 2010), leakage, and migration from the injection site (Ballios et al., 2010).

Evidence to date suggests that transient encapsulation of cells for delivery enhances their survival under the adverse conditions within the disease or injury-damaged host target organs (Karoubi et al., 2009; Mayfield et al., 2014). Transplantation of RPCs along with a supporting three-dimensional scaffold has indeed been shown to improve cell survival, retention, differentiation, organization (Hynes and Lavik, 2010), and integration (Ballios et al., 2010; Hynes and Lavik, 2010).

A range of biomaterials have been evaluated as scaffolds for tissue engineering in general, and the retina in particular. The most promising scaffolds for cell delivery are hydrogels that emulate the extracellular matrix (ECM), and specifically, injectable hydrogels that will deliver RPCs to the target site. While the use of ECM macromolecules and other biomaterials are promising, such as hyaluronic acid and methylcellulose (Ballios et al., 2010), the safety and efficacy of these materials will need to be validated before they can be evaluated in humans. Biocompatibility and immune compatibility are critical considerations in the development of any biomaterials-enabled therapy. Thus, a therapy that relies on biomaterials that originates from each patient would therefore mitigate these issues.

In studies on RPE transplantation, adhesion to ECM components can be improved through enhancing integrin function (Fang et al., 2009; Afshari et al., 2010), suggesting that targeting integrin-based adhesion might also be a strategy to modulate retinal progenitor survival and differentiation. Thus in this study, we have focused our research on fibrin glue (FG), since it can be isolated autologously in a crude form from patients, and fabricated into a hydrogel scaffold without the potential risk of foreign body reaction or infection (Ahmed et al., 2011) and would promote integrin-based adhesion. With the advent of induced pluripotential stem cells, it may become possible to also use autologous cells from the patients to avoid immune compatibility effects as recent developments in iPS-derived retinal cells and tissues (Cyranoski, 2014; Kamao et al., 2014; Reichman et al., 2014). We have previously examined the use of FG (extracted from patient plasma through apheresis and processed to obtain fibrinogen and thrombin) as a scaffold for use in cartilage tissue engineering, in combination with bone marrow-derived human mesenchymal stem cells (BM–hMSCs) (Ahmed et al., 2011). In the eye, human fetal retinal pigment epithelial (HFRPE) cells have been grown on crosslinked fibrin microsphere for subretinal delivery into a rabbit model, where they survived for over 1 month (Oganesian et al., 1999). Here, we evaluated the use of a cell delivery system comprising autologous FG. This is produced by cryoprecipitation of fibrinogen and thrombin from patient plasma, with subsequently mixing to form a fibrin sealant. We hypothesized that transient encapsulation of RPCs within the FG preparation could promote enhanced proliferation and differentiation.

## Materials and Methods

### Materials

Fibrin glue was obtained autologously as described below. The peptides, GRGDSP and GRGESP (sterile-filtered HPLC grade), were purchased from Bachem Bioscience, Inc. (Torrance, CA, USA) while RGDS (sterile-filtered HPLC grade) was purchased from Tocris Bioscience, Inc. (Bristol, UK). Bromodeoxyuridine (BrdU) reagent was purchased from Roche Applied Science (Mississauga, ON, Canada). Tissue culture media, L-glutamine, and penicillin/streptomycin were purchased from Invitrogen (Life Technologies, Burlington, ON, Canada). All other materials were obtained from Sigma-Aldrich (Oakville, ON, Canada).

### Fibrin glue production

With ethical approval from the Ottawa Hospital Research Ethics Board, and written, informed consent, 250 mL of pooled human plasma was collected from two healthy donors by apheresis using the Trima Accel Version 5 automated blood collection system (Gambro BCT, Lakewood, CO, USA). Other blood components, such as cells, were returned to the circulation (Ahmed et al., 2011). FG is composed of two components – fibrinogen and thrombin. Cryoprecipitates of fibrinogen and thrombin were obtained using the CryoSeal^®^FS System and associated Thrombin Processing Device™, respectively (ThermoGenesis Corp., Rancho Cordova, CA, USA). These devices produce cryoprecipitated fibrinogen at a concentration of ~20.1 mg/mL, and thrombin at ~38 U/mL. Each component was aliquoted and stored frozen at −80°C until used (Ahmed et al., 2011).

### Retinal progenitor cell isolation and culture

Animal work was performed in accordance to the Association for Research in Vision and Ophthalmology (ARVO) Statement for the Use of Animals in Ophthalmic and Vision Research. Mouse RPCs were isolated and cultured as previously described (Ringuette et al., 2014). Briefly, eyes of euthanized neonatal C57BL/6 mice at postnatal day 0 (PN0) or day 1 (PN1) were dissected in CO_2_-independent DMEM medium. The sclera, lens, and retinal pigment epithelium were removed, and the remaining neural retinas were transferred onto 13 mm polycarbonate filters (0.8 μm pore size, Nucleopore). The explants were flattened and cultured with the ganglion cell layer facing up within 24-well plates at 8% CO_2_ and 100% humidity. Each well was supplemented with 0.5 mL of serum-free retinal cell culture medium [SFRCM; DMEM/F12 (1:1)], 10 μg/mL insulin, 100 μg/mL transferrin, 100 μg/mL bovine serum albumin (BSA Fraction V), 60 ng/mL progesterone, 16 μg/mL putrescine, 40 ng/mL sodium selenite, 25 μg/mL gentamicin, and 20 nM *Smoothened* agonist (Hh agonist) (Frank-Kamenetsky et al., 2002), a gift from Curis Inc., Lexington, MA, USA. After 2 days, the retinal explants were digested in 1 mL of trypsin solution (0.75 μg/mL, Sigma) at 37°C for 10 min. The digestion was stopped by the addition of 1 mL trypsin inhibitor (1 mg/mL in SFSCM) followed by trituration to single cells. The cell suspension was centrifuged at 1500 rpm for 5 min and the pellet was re-suspended in serum-free stem cell culture medium [SFSCM; DMEM/F12 (1:1), 6 ng/ml progesterone, 5 ng/mL selenium, 100 μg/mL transferrin, 9.5 μg/mL putrescine, 250 μg/mL insulin, 25 ng/mL human epidermal growth factor (EGF), 10 ng/mL fibroblast growth factor 2 (FGF-2), and 2 μg/mL heparin (Tropepe et al., 2000) supplemented with 5 nM Hh agonist]. The cells were cultured in 6-well plates at the density of 5–10 × 10^5^ cells per well in 2 mL of SFSCM or in 24-well plates at a density of 5–10 × 10^4^ cells per well in 0.5 mL of SFSCM. The medium was refreshed every 2 or 3 days. After 2 weeks, a monolayer formed and the cultures were passaged every 2–3 days by mechanical trituration to obtain single cells that were then diluted 1:3 with fresh culture medium. Differentiated neurons did not survive under these culture conditions and were lost upon serial passaging of the cultures (Ringuette et al., 2014). Seven batches of RPCs were used during the whole study.

### Encapsulation of retinal progenitor cells in fibrin glue

**Figure 1:**
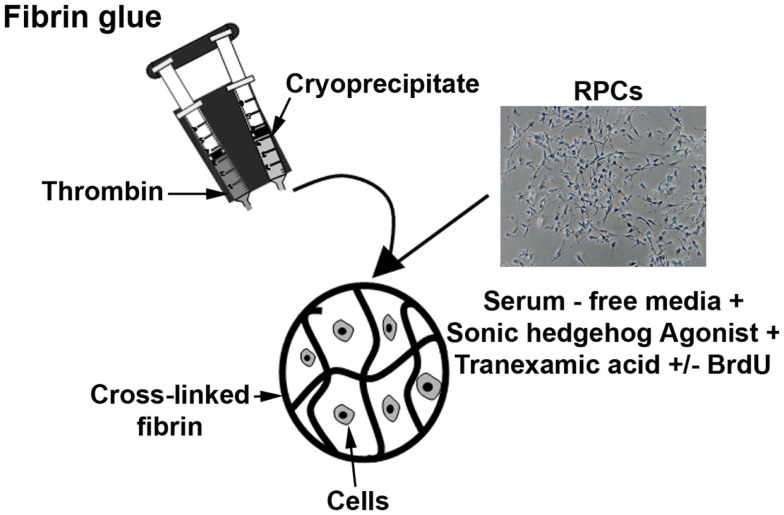
**Diagram showing the preparation of fibrin-encapsulated RPCs**. Aliquots of cryoprecipitated fibrinogen and thrombin, and retinal progenitor cells were combined and allowed to polymerize (with thrombin serving as a cross-linker). The encapsulated cells were then cultured in serum-free media with additives such as sonic hedgehog agonist, tranexamic acid, and BrdU.

Figure 1 summarizes the preparation of fibrin-encapsulated RPCs. Aliquots of cryoprecipitated fibrinogen (42.5 μL) and 15 μL cultured RPCs were gently but thoroughly mixed. Polymerization was initiated by mixing in of 42.5 μL of thrombin (from same unit as cryoprecipitate) to a final volume of 100 μL of gel. Final concentrations of fibrinogen, thrombin, and cells within each gel were ~8 mg/mL, ~13 U/mL, and 10^7^ cells/mL, respectively. Resulting hydrogels were disk-shaped constructs 6.5 mm diameter and 2 mm thick. After incubation for 30 min at 37°C to allow optimum coagulation, gels were cultured in Transwell^®^ culture inserts and supplemented with SFSCM medium containing 5 nM Hh agonist and 1.5 mg/mL tranexamic acid (protease inhibitor). Implants were maintained at 37°C in a humidified incubator with 5% CO_2_ for up to 7 days with media changed every 3–4 days. In the absence of protease inhibitors, fibrin gels completely degraded over 2–3 days in culture; however, the gels were fully stabilized by addition of tranexamic acid to the medium. At 0, 1, 3, and 7 days, gels were collected for immunohistochemical evaluation or RNA isolation.

### Immunohistochemistry

Fibrin glue-RPC constructs (FG-RPCs) were examined for the expression of integrin α7, which is upregulated when cells bind to ECM macromolecules, SRY related HMG BOX gene 2 (SOX2), which marks RPCs (Steedman et al., 2010), and photoreceptor-specific markers – recoverin (RC), cone-rod homeobox (CRX), and rhodopsin, which is found only in mature rod photoreceptors.

Fibrin hydrogels containing RPCs were fixed in 4% paraformaldehyde for 30 min at room temperature, cryoprotected in 30% sucrose for 24 h, and then frozen in 1:1, 30% sucrose: OCT and stored at −80°C. Cryosections of 10 μm thickness were placed onto slides and stored at −80°C. Cultured monolayers of cells were fixed in 4% paraformaldehyde for 30 min at 4°C then washed two times in 0.1 M phosphate buffered saline (PBS; pH7.4), and stored at 4°C. For immunohistochemistry, slides with frozen sections were equilibrated to room temperature and washed three times in PBS (5 min each) prior to incubating in blocking buffer (PBS containing 5% heat-inactivated fetal bovine serum and 1% bovine serum albumin) for 1 h to prevent non-specific antibody binding. After blocking, slides were washed again in PBS, and then incubated overnight at 4°C in one of the following primary antibodies: SOX2, RC, CRX, rhodopsin, and integrin α7, prepared in blocking buffer plus 0.3% Triton X-100 (PBST). Details of the antibodies and dilutions used are listed in Table 1. The next morning, the slides were washed three times in PBS and incubated with the secondary antibody prepared in PBST for 1 h at room temperature. After a further two washes in PBS, the slides were stained for 3 min with DAPI stain (1:10,000 in PBS), washed again twice in PBS, and cover-slipped with PBS for visualization using a Nikon Eclipse TE2000-E microscope (Nikon Instruments, Inc.) and accompanying software (NIS-Elements™ AR3.10). For negative controls, the primary antibody was omitted. For positive controls of SOX2 and RC (early RPC markers), PN0 mouse eyes were used, while for CRX and rhodopsin (late stage marker) and integrins, adult mouse eyes served as positive controls.

**Table 1:** **Antibodies used for immunohistochemistry**.

No.	Antibody	Host	Type	Final concentration	Company and catalog number
1	Anti-BrdU antibody	Mouse	Monoclonal	1:500	A21300, Invitrogen
2	Anti-SOX2 antibody	Rabbit	Polyclonal	1:200	AB5603, Millipore
3	Anti-RC antibody	Rabbit	Polyclonal	1:500	AB5585, Millipore
4	Anti-CRX antibody	Rabbit	Polyclonal	1:50	Gift from Dr. C. Gregory-Evans
5	Anti-rhodopsin antibody	Mouse	Monoclonal	1:50	B630, Developmental Studies Hybridoma Bank
6	Anti-integrin α7 antibody	Mouse	Monoclonal	1:50	K0046-3, MBL International Company
7	Anti-mouse IgG (H + L) antibody, Alexa Fluor^®^488 (green)	Goat	Polyclonal	1:400	A11029, Invitrogen
8	Anti-rabbit IgG (H + L) antibody, Cy3™ conjugate (red)	Goat	Polyclonal	1:500	111-165-003, Jackson ImmunoResearch Laboratories

### Effects of fibrin and tranexamic acid on cell viability and proliferation

The effects of fibrin and tranexamic acid on cell viability and proliferation were examined. To label proliferating cells, RPC encapsulated in fibrin hydrogels were prepared and cultured as described above. Ten μM BrdU was added to the culture medium to label cells in S-phase. For BrdU staining of cells or cyro-sectioned material, slides were equilibrated to room temperature, washed three times in PBS (5 min each), and then incubated in 1N HCl for 10 min on ice, followed by incubation in 2N HCl for 10 min at room temperature and then 20 min at 37°C. Immediately after the acid washes, the slides were treated with 0.1 M borate buffer (pH 8.5) for 12 min at room temperature then washed twice in PBS (5 min each). The sections were incubated with anti-BrdU primary antibody (as per Table 1) and then processed as described above. In order to determine the viability of encapsulated RPCs, apoptotic cells were detected using the ApopTag^®^ Fluorescein *in situ* apoptosis detection kit (S7110; Millipore) following the manufacturer’s protocol.

### Adhesion of retinal progenitor cells to fibrin

Two major classes of adhesion sites have been identified in the fibrin (ogen) molecule. One of them is mediated by RGD sequences within the α chain of fibrinogen, while the other site is RGD-independent (Chernousov and Carey, 2003). To identify the site that was directing the adhesion and spreading of RPCs onto fibrinogen, cells were plated on fibrinogen-coated plates (Chernousov and Carey, 2003) and maintained in the previously mentioned culture media containing different concentrations (0, 10, 25, 50, and 100 μM) of either of the RGD peptides (GRGDSP and RGDS) and compared to cells plated on fibrinogen in the presence of a control non-RGD peptide (GRGESP). Data were expressed as mean ± SE. A two-way analysis of variance (ANOVA) for RPCs binding to FG-coated plates in the presence of different peptides was performed using Stata Statistical Software (StataCorp LP, Release 11, 2009. College Station, TX, USA). To make the most conservative conclusions regarding significance of the results, Bonferroni *post hoc* tests were performed, as this is a very stringent test to compare means of the different study groups. Results were considered significant when ANOVA was confirmed by Bonferroni *post hoc* test at *p* < 0.05.

### Real-time polymerase chain reaction

Mouse-specific primers for *SOX2*, *CRX*, *integrin* α*7*, *integrin* β*3*, and the control *glyceraldehyde-3-phosphate dehydrogenase* (*GAPDH*) were obtained from SABioscience (Qiagen Inc., Mississauga, ON, Canada) and are described in Table 2. RNA was extracted from fibrin-encapsulated RPCs at different time points using the RNeasy Fibrous Tissue Kit (Qiagen) and following the manufacturer’s protocol. First-strand cDNA was synthesized using ThermoScript™ RT-PCR system for first-strand cDNA synthesis kit as per manufacturer’s instructions. Polymerase chain reaction amplification and melt curve analysis were performed using a BioRad iCycler thermocycler as previously described (Ahmed et al., 2011).

**Table 2:** **Primers used for PCR**.

Target gene	Gene symbol	Accession no.	Annealing T (°C)	Band size (bp)	Catalog number
Glyceraldehyde-3-phosphate dehydrogenase	GAPDH	NM_008084.2	60	140	330001-PPM02946E
SRY-box containing gene 2	SOX2	NM_011443.3	60	110	330001-PPM04762E
Cone-rod homeobox containing gene	CRX	NM_007770.4	60	191	330001-PPM25460A
Integrin alpha 7	ITGA7	NM_008398.2	60	182	330001-PPM03611E
Integrin beta 3	ITGB3	NM_016780.2	60	67	330001-PPM03687E

The results were analyzed using the comparative CT method. Monolayer RPCs culture was used as a baseline for each gene, which was normalized to GAPDH housekeeping expression. Data were used to compare gene expression at 0, 1, 3, and 7 days with triplicate determinations of four independent cultures (*n* = 4). The ΔΔC_T_ was calculated using the equation: ΔΔC_T_ = ΔC_T(target)_ − ΔC_T(baseline)_. The relative gene expression level was determined by calculating 2^(−ΔΔCT)^. The identity of the DNA products obtained at the end of real-time PCR cycles was confirmed using 4% agarose gel and GelGreen™ DNA dye by comparing to data stated in the manufacturer’s protocol. Data were expressed as mean ± SE.

## Results

### Effects of FG and tranexamic acid on cell viability, proliferation, and differentiation

**Figure 2:**
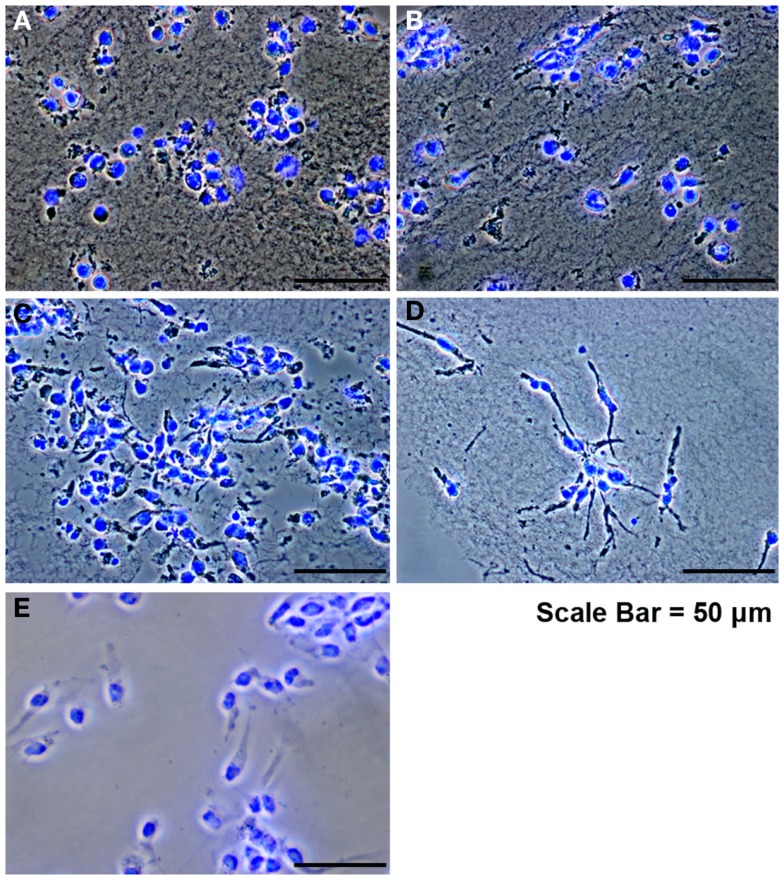
**Retinal progenitor cells cultured in fibrin hydrogels**. **(A)** RPCs after encapsulation, showing an initial rounded morphology. **(B–D)** RPCs at days 1, 3, and 7 showing attachment and spread. **(E)** Control RPCs cultured as monolayer on tissue culture plastic.

**Figure 3:**
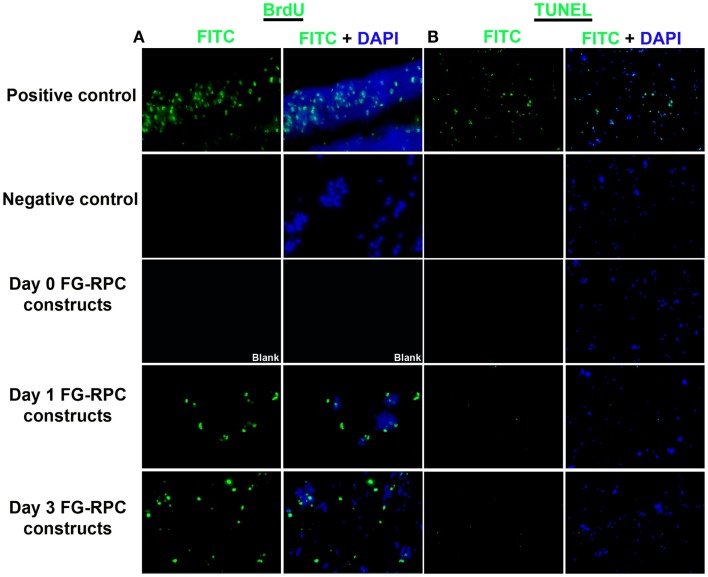
**(A)** Localization of proliferating retinal progenitor cells that have incorporated BrdU; **(B)** localization of apoptotic cells using the TUNEL assay.

Since the main problem of subretinal injection of RPCs is the poor cell survival (Tomita et al., 2005; Ballios et al., 2010), the effect of FG encapsulation on RPCs survival and proliferation, in the presence of tranexamic acid, was investigated in this study. The 7-day time course was chosen based on the differentiation kinetics of perinatal rodent precursor cells *in vitro* (Kelley et al., 1994; Morrow et al., 1998). Within the fibrin capsules, RPCs initially had a rounded morphology (Figure 2A). However, over the 7-day culture period, the cells moved out of the capsules and attached to the substratum (Figures 2B–D), where they assumed a more spread-out morphology, similar to that of cells in monolayer cultures (Figure 2E). The BrdU assay results showed that neither fibrin-encapsulation nor the addition of tranexamic acid to the culture medium had any effect on RPC proliferation (Figure 3A). Conversely, TUNEL assay results showed that neither fibrin-encapsulation nor addition of tranexamic acid to the culture medium resulted in any increase in incidence of apoptosis (Figure 3B).

**Figure 4:**
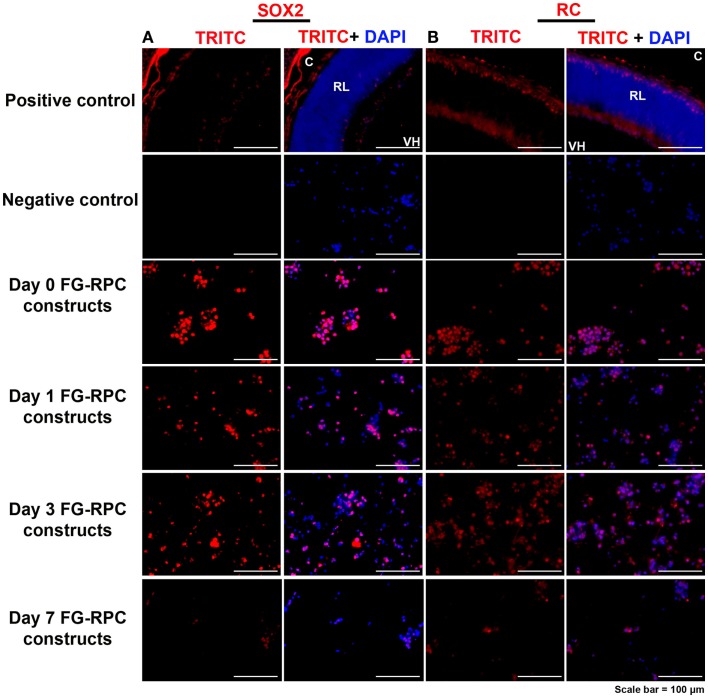
**Expression of (A) SOX2, which is expressed in early retinal progenitor cells, and (B) recoverin, which is expressed by presumptive photoreceptors (cones) in the developing retina, by retinal progenitor cells encapsulated in fibrin glue (FG) at different time points after placing in cell culture**. C, choroid; RL, retinal layers; VH, vitreous humor. Negative controls comprise omission of the primary antibody; positive controls comprise sections through postnatal day 0 mouse eyes.

**Figure 5:**
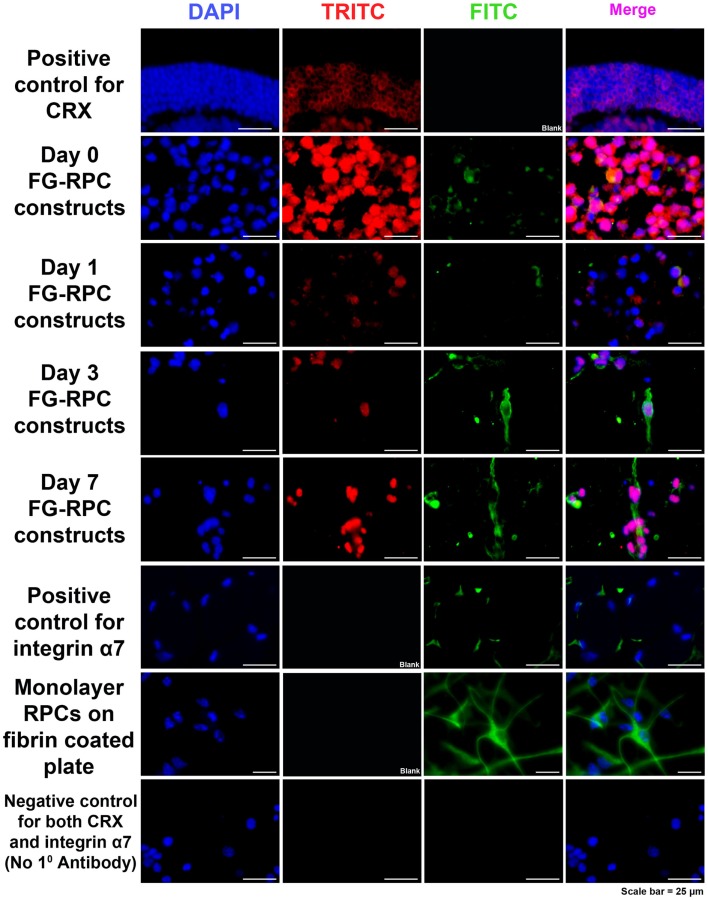
**Immunohistochemistry, showing positive staining for CRX (red) and integrin α7 (green) in RPCs after encapsulation into FG at the different time points**. Positive controls for CRX are cryosections of adult mouse eyes. Positive control for integrin α7 are adult mouse orbital muscle sections. Monolayer RPCs attached to fibrin-coated plates serve as a second positive control for integrin α7. The negative control for CRX and α7 are immunostained cryosections of FG-RPC constructs with the omission of the primary antibody.

Immunohistochemistry performed on FG-encapsulated RPCs compared to non-encapsulated controls to compare the effects of the encapsulation on cell behavior, shows that the retinal stem cell markers were retained early on during the culture. Positive staining for both SOX2 and RC, a photoreceptor marker, was observed in sections of FG-RPCs constructs at days 0, and 3 of culture (Figure 4). However, by day 7, expression of SOX2 and RC has dropped of considerably (Figure 4). RPCs encapsulated within fibrin were positive for CRX at day 0 (Figure 5). The expression on days 1 and 3 appears to be weaker but was strong again by day 7. Integrins are transmembrane proteins that modulate cell adhesion, proliferation, and differentiation. They initiate cell signaling pathways upon interaction with the ECM (Welser et al., 2007). We identified two integrins that were expressed by RPCs when plated onto fibrin-coated plates, α7 and β3. The expression of integrin α7 was weak initially, but became more pronounced on days 3 and 7. The RPCs that were seeded as monolayers on top of a fibrin substrate showed strong immunostaining for integrin α7 (Figure 5). Rhodopsin, which is only expressed by mature photoreceptors, however, was negative at all time points, in both encapsulated and non-encapsulated RPCs (data not shown). These results indicated that contact of RPCs with FG induced cell spreading and improved expression of two integrin subunits (α7 and β3) and early (CRX and RC) but not late stage differentiation markers (rhodopsin).

### RPC adhesion to FG

**Figure 6:**
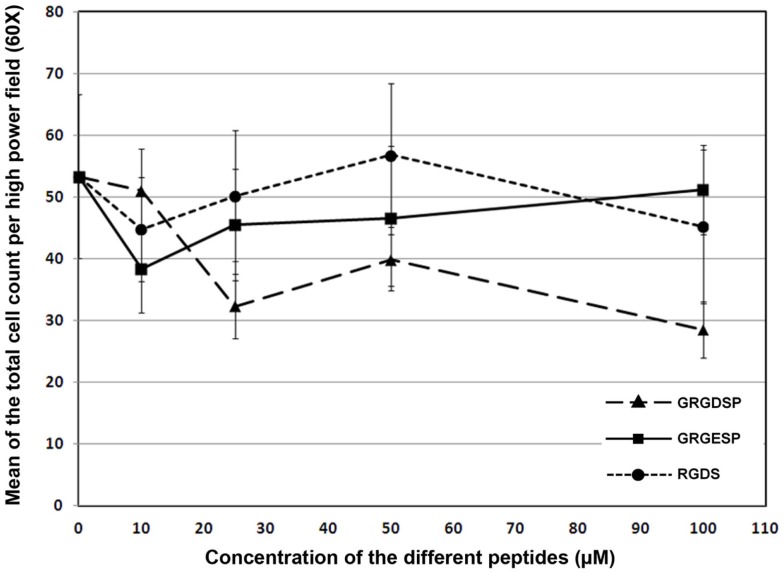
**Line chart showing the effect of different RGD peptides (GRGDSP and RGDS) on the attachment of RPCs to fibrin-coated plates as compared to a control peptide (GRGESP)**. Data were expressed as mean ± SE (*n* = 6). Results were considered significant when ANOVA was confirmed by Bonferroni *post hoc* test at*p* < 0.05.

There was no significant change in the mean value of numbers of attached and spread cells on fibrin substrates with the introduction of increasing concentrations of competing RGD peptides GRGDSP or RGDS (*p* = 0.18, *F* = 1.77). The line graph indicated that adhesion and spreading of RPCs onto fibrin – coated plates in the presence of RGD peptides were comparable to that of cells plated in the presence of the control non-RGD peptide. As high as 100 μM of the aforementioned RGD peptides were unable to inhibit RPCs adhesion and spreading onto fibrinogen-coated plates when compared to cells cultured on fibrinogen-coated plate in the presence of comparable concentration of non-RGD peptide (Figure 6), suggesting that the binding of RPCs to the FG substrate is most likely through a mechanism that is independent of RGD.

### Effect of FG on RPC phenotype

**Figure 7:**
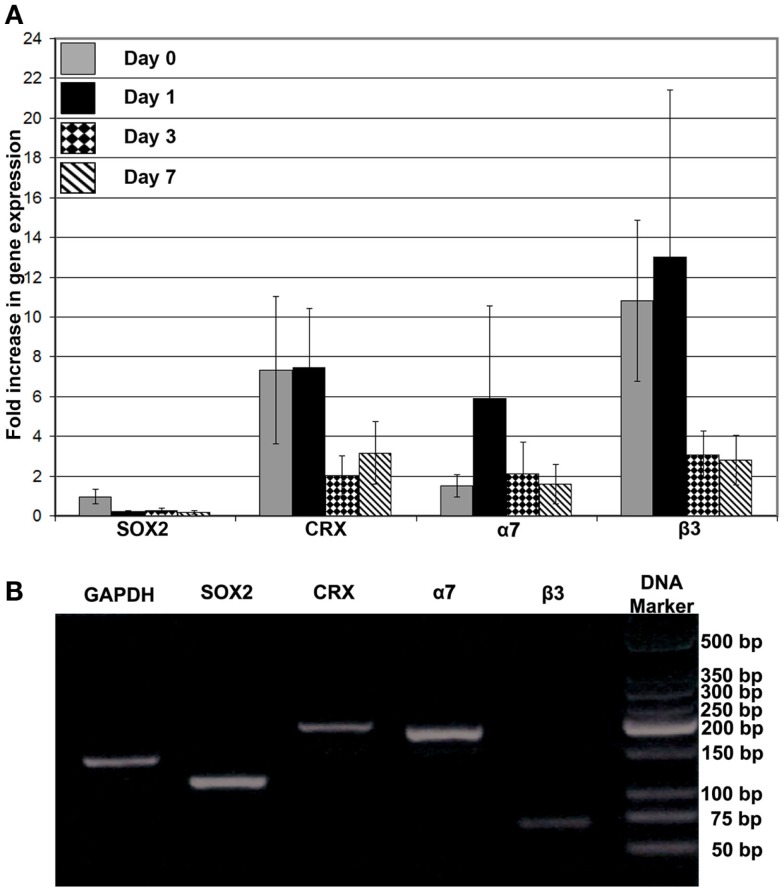
**(A)** Gene expression analysis of *SOX2*, *CRX*, *integrin* α*7*, and *integrin* β*3* after encapsulation of RPCs into FG at 0, 1, 3, and 7 days; **(B)** DNA electrophoresis of the real-time PCR products for the different indicated genes. Monolayer RPCs culture was used as a baseline for each gene, which was normalized to GAPDH housekeeping expression. Data were expressed as mean ± SE (*n* = 4)

The effects of FG on the phenotype of encapsulated RPCs was characterized by examining the expression of markers for progenitor cells versus early differentiation (SOX2 and CRX, respectively), and expression of transmembrane receptors that mediate cell–ECM interactions (integrin subunits α*7* and β*3*). Directly after encapsulation of RPCs into FG (i.e., day 0), *SOX2* gene expression did not show noticeable change compared to expression in monolayer RPCs culture. However, over the ensuing week, SOX2 gene expression declined compared to day 0 construct. In contrast, encapsulation of RPCs into FG resulted in sevenfold initial increase in CRX expression (days 0 and 1). However, this enhanced expression was diminished at later time points – day 3 and day 7 (two and threefold, respectively). We evaluated the gene expression of two integrins, α*7* and β*3* subunits. The encapsulation of RPCs into FG resulted in no increase in integrin α*7* gene expression directly after encapsulation into FG (day 0). A sixfold increase was observed at 24 h post encapsulation (day 1). However, this increased α*7* gene expression did not last, as expression decreased again at days 3 and 7 (twofold increase at both time points). Integrin β*3* expression showed a marked 11- and 13-fold increase at days 0 and 1, respectively. However, the expression was decreased to threefold at day 3 and day 7 time points (Figure 7).

## Discussion

### Biocompatibility of FG-tranexemic acid scaffolds

Our results showed that encapsulation of RPCs with fibrin precursors that can be potentially isolated from autologous sources allows the cells to proliferate and differentiate, albeit, not to full maturity *in vitro*. Encapsulation of RPCs into FG did not affect cell proliferation, nor was it cytotoxic in our study. The absence of cytotoxic effects in our system is consistent with previous studies that reported the safety of FG when used with the different retinal cell types or implanted into different ocular regions (Robinson and Madison, 2000; Pardue et al., 2004). However, it should be noted that not all FG is similar, as the use of xenogeneic FG (porcine glue in rabbits with human retinal pigment epithelium cells), caused acute inflammation that resulted in fibrosis (Hou et al., 2009). Nevertheless, FG has been successfully used as an adhesive substrate for attachment of peripheral nerve grafts to optic nerve stumps, and is permissive for retinal ganglion cell axon regeneration (Robinson and Madison, 2000). Similar studies establishing the safety of FG and fibrin-encapsulation have been reported for a range of cell types, including bone marrow mesenchymal stromal cells (Ahmed et al., 2011), chondrocytes (Meinhart et al., 1999), carotid artery derived cells (smooth muscle cells/fibroblast; Cholewinski et al., 2009), and human neural progenitor cells (Cox et al., 2003).

Tranexamic acid is an antifibrinolytic agent that is used clinically to control bleeding by preventing clotted blood from breaking down. As FG is essentially a “blood clot,” tranexamic acid was evaluated for its capacity to stabilize the constructs that on their own were unstable in culture medium. The addition of tranexamic acid stabilized the fibrin scaffolds without adversely affecting RPC survival or proliferation. Hence, the combination of FG and tranexamic acid appears to be a suitable as a cell encapsulation and delivery vehicle.

### Proliferation and differentiation of RPCs in FG

While the induction of RPCs from iPS and ES cells has been demonstrated, optimizing photoreceptor differentiation in these cultures remains a challenge (Lamba et al., 2006; Lamba et al., 2010; Assawachananont et al., 2014). Principals that guide photoreceptor differentiation from RPCs could be exploited to optimize photoreceptor differentiation from a variety of SC sources. RPCs are undifferentiated, proliferative cells, characterized by the expression of SOX2, a neural progenitor marker (Klassen et al., 2007; Steedman et al., 2010), and the absence of CRX, a marker of post-mitotic photoreceptors and rhodopsin, a late stage marker of rod photoreceptors (Swaroop et al., 2010). FG-encapsulated RPCs showed SOX2 and RC expression at days 0 and 3 of culture. SOX2 but not RC was expressed by RPCs grown as monolayer cultures in our previous studies (Ringuette et al., 2014). The difference between the two studies could be due to the low levels of growth factor(s) that would have been incorporated into the FG that was not present in the RPC culture medium. FG-encapsulated RPCs lost SOX2 expression and gained CRX expression over time in the cultures, a result that was corroborated by immunostaining and gene expression analysis. The inverse relationship between the differentiation marker CRX and the progenitor marker SOX2 observed in our study is consistent with earlier studies which revealed that up-regulation of fate-specific markers including CRX, RC, and rhodopsin (Steedman et al., 2010; Swaroop et al., 2010) indicates enhanced differentiation of the RPCs toward rod cell fates. Differentiation of progenitors is accompanied by down-regulation of SOX2 (Steedman et al., 2010). These results suggest that the culture environment, possibly the attachment of the cells to the fibrous scaffold induced a differentiation program in these cells. This result is consistent with evidence in other studies that the 3D ECM-like environment promotes RPC differentiation (Steedman et al., 2010).

A wide range of growth factors are present in trace amount in the crude form of FG including platelet-derived EGFs, platelet-derived growth factor A + B (PDGF-AA, AB, BB), TGFα1, TGF-β2, insulin-like growth factor 1 and 2 (IGF1 and 2), vascular endothelial growth factor (VEGF), and basic FGF-2 that are released from the platelets (exist in low amount in crude FG) upon activation by thrombin. In addition to these bioactive factors, exogenous growth factors were added to the culture medium including EGF, progesterone, and Smoothened agonist (Hh agonist). Growth factors can either stimulate or inhibit cellular processes such as division, migration, differentiation, and gene expression, depending on the cells involved. Therefore, after encapsulation of stem cells, the combination of growth factors in FG would have a complex effect on their cellular activity and patterns of gene expression (Ahmed et al., 2011). Here, the RPCs initiated differentiation but did not achieve terminal differentiation. It is possible that *in vivo* grafting may enable the cells to terminally differentiate. RPCs on their own have failed to engraft or differentiate into retinal neurons, when transplanted *in vivo* into mouse eyes (Ringuette et al., 2014). Hence, this differentiation promoting effect of FG on RPCs, on its own, or together with incorporated modulatory bioactives, warrants further investigation as it could be exploited in the context of retinal cell transplantation.

### RPC–FG scaffold interaction

Another crucial aspect of retinal tissue engineering is cell attachment to the scaffold surface. To implant RPCs successfully into the subretinal space, cells must be firmly attached to the scaffold surface and be able to resist shear forces that occur during implantation. It has been demonstrated that protein modification of polymeric scaffold can induce attachment of progenitor cells, which subsequently promotes the differentiation process (Steedman et al., 2010). The present study showed that RPCs exhibited a spread-out morphology after encapsulation, indicating adhesion and attachment of the cells to FG. This observed attachment is accompanied by up-regulation of the integrin α7 and β3 genes. Positive immunostaining for integrin α7 protein was also observed at the different time points that were consistent with α7 gene expression. Integrin α7 can bind fibrin (Hinds et al., 2011) and is thought to be essentially a muscle cell specific receptor (Basora et al., 1997), as it is a major integrin expressed in skeletal, cardiac, and smooth muscle (Mielenz et al., 2001). However, non-muscle locations of integrin α7 expression have been also identified, including the vasculature, intestinal epithelial cells (Basora et al., 1997), and nervous system (Velling et al., 1996). It has been also shown that integrin α7 expression in sensory neuron subtypes can be induced following nerve injury in mice and mediates neurite outgrowth (Gardiner et al., 2005). In addition, integrin is an important mediator of axonal outgrowth and regeneration. Integrin α7 is one of the prevalent integrin subunit on neurons and axons in the developing avian spinal cord (Kil and Bronner-Fraser, 1996) and is concentrated on growth cones in the regenerating nerve (Werner et al., 2000). Given the precedent for α7 in the nervous system, its expression in the FG-encapsulated RPCs is to be expected. With respect to integrin β3, studies of normal human retina and choroid have demonstrated the presence of this subunit in retinal and choroidal blood vessels. In addition, integrin β3 was expressed in the normal human optic nerve (Elner and Elner, 1996) and hence, expression may also be expected in RPCs.

It has been shown that fibrin (ogen) mediates attachment and spreading of many cell types (Chernousov and Carey, 2003). The lack of inhibitory effect of the two competing RGD peptide on RPC adhesion to fibrinogen suggests that the attachment of RPCs to fibrinogen is mediated by a RGD-independent mechanism. This result is consistent with the evidence for RGD-independent adhesion of integrins α7 and β3 to ECM proteins (Ruoslahti, 1996; Hynes, 2002; Barczyk et al., 2010; Joddar and Ito, 2011). However, it has also been shown that β3 also binds to many ECM proteins through the RGD sequence (Ruoslahti, 1996; Joddar and Ito, 2011). It has been shown that α7 integrin is an important laminin receptor and is involved in the formation of the neuromuscular and myotendinous junctions (Ozeki et al., 2006). In addition, a number of laminin chains have been shown to be expressed in retinal basement membranes (Edwards and Lefebvre, 2013). Therefore, the α7 integrin binding proteins, laminins, are available to normal RPCs *in vivo*, which might explain the capability of RPCs to express α7 integrin in the context of FG.

## Conclusion

In conclusion, FG is a potential encapsulating material for delivery of RPCs into the subretinal space in terms of cell proliferation and survival. FG promoted the adhesion and initiated the differentiation of RPCs after encapsulation. Adhesion of RPCs to FG was mediated by a RGD-independent mechanism. Optimization of FG-assisted RPC delivery into the subretinal space will be needed for further translational efforts. The combination of FG with bioactive factors such as activin A, taurine, or retinoic acid (Kicic et al., 2003; Qiu et al., 2007) that promote differentiation of RPCs; or with incorporated cell adhesive peptides that modulate cell attachment such as DGEA, RGD,YGYYGDALR, IKVAV, YIGSR, or RNIAEIIKDI (Ahmed et al., 2008; Joddar and Ito, 2011) would also be important avenues to explore.

## Conflict of Interest Statement

The authors declare that the research was conducted in the absence of any commercial or financial relationships that could be construed as a potential conflict of interest.
